# Combating coronavirus disease (COVID-19) in rural areas of Malawi: Factors affecting the fight

**DOI:** 10.4102/phcfm.v15i1.3464

**Published:** 2023-01-30

**Authors:** Winner Chawinga, Wales Singini, John Phuka, Nathaniel Chimbatata, Collins Mitambo, Clara Sambani, Dzinkambani Kambalame

**Affiliations:** 1 Department of Information Sciences, Faculty of Humanities and Social Sciences, Mzuzu University, Mzuzu, Malawi; 2 Research Directorate, Mzuzu University, Mzuzu, Malawi; 3 School of Public Health and Family Medicine, Kamuzu University of Health Sciences, Blantyre, Malawi; 4 Department of Nursing and Midwifery, Faculty of Health Sciences, Mzuzu University, Mzuzu, Malawi; 5 Public Health Institute of Malawi, Ministry of Health, Malawi, Lilongwe, Malawi; 6 Research Department, Malawi Ministry of Health, Lilongwe, Malawi

**Keywords:** COVID-19, crowded places, face masks, social distancing, Malawi

## Abstract

**Background:**

Owing to their detachment from urban areas, people living in rural areas of Malawi are on the receiving end of health services and socio-economic benefits. The study therefore explored how Malawians living in these areas are adhering to coronavirus disease 2019 (COVID-19) containment measures and the factors that affect the COVID-19 fight amongst this population.

**Aim:**

The study investigated how the rural population in Malawi adheres to COVID-19 containment measures.

**Setting:**

The study was conducted in two districts in Northern Malawi.

**Methods:**

Data were collected from 263 participants. The chi-square (*χ*^2^) test was performed to determine the association between demographic variables and COVID-19 prevention practices and factors affecting the COVID-19 fight.

**Results:**

Education was significantly associated with wearing of masks (*p* = 0.01), use of sanitisers (*p* < 0.01) and practising social distancing (*p* = 0.07). Monthly income was associated with the use of sanitisers (*p* < 0.01). Women were more exposed to fake news about COVID-19 (*p* = 0.09); older people were more likely to disregard COVID-19 containment measures for cultural reasons (*p* = 0.07); and monthly income was associated with a lack of resources for following COVID-19 containment measures (*p* < 0.01).

**Conclusion:**

Findings show that factors affecting the COVID-19 fight are influenced by various socio-economic factors which should therefore be taken into account in policy planning aiming at controlling the pandemic.

**Contribution:**

The study provides health stakeholders with a policy direction that enhances better coordination, implementation and monitoring of COVID-19 response and recovery activities in rural areas in Malawi. The findings have implications on controlling current and future communicable diseases; the proposed strategies might be employed in fighting similar current and future pandemics.

## Introduction

Despite some milestones registered in the coronavirus disease 2019 (COVID-19) fight, such as breakthroughs in vaccine discoveries, the pandemic is still threatening millions of lives across the globe. Some research that compared the coronavirus to the seasonal flu established that it is more contagious and deadly, with the median reproductive index (R_0_) of 2.2 for coronavirus compared to 1.28 of seasonal flu.^1,2^ This implies that a person with the flu virus may only transmit the infection to another person, while the same person if infected with coronavirus may infect two or more different people.^3^ As predicted by Eubank and others^4^ in the early days of the pandemic that it might claim tens of millions of people’s lives, statistics show that the pandemic has infected millions of people.^5^ The pandemic has therefore put unprecedented pressure on the global health system, hence compelling governments and health stakeholders to come up with desperate health policies that can contain the worst health crisis in humankind’s history.^3^

A recent study has reiterated that change in human behaviour is the most effective strategy to mitigate the spread of the pandemic.^6^ Such individual behavioural changes recommended by the World Health Organization (WHO) include avoidance of public gatherings, practising social distancing, washing hands frequently and wearing masks.^7,8,9^ Other scholars have made it clear that while biomedical practice is key in disease diagnosis and treatment, infection prevention and control (IPC) is fundamentally a behavioural science.^10^ However, research has shown that the implementation of these behavioural changes has been met with resistance in most countries. In the United States of America, for example, Americans perceive social distancing, quarantining and even mask-wearing as a violation of their freedom and personal liberty.^11^ Malawi may not be immune to such challenges; hence, it becomes necessary to assess the challenges that confront the fight against COVID-19, especially considering the prevailing complex cultural diversity and economic disparities in the country.

Being part of the larger global world, Malawi has not been spared from the COVID-19 pandemic.^12^ The first case was confirmed on 02 April 2020, and new cases of infection and death have been recorded almost daily. Sensing the devastating threat that came along with the pandemic, the Malawi government enforced strict containment measures, including a 21-day national lockdown, which was, however, immediately set aside by the courts on the basis that it was enforced without consideration of people’s economic hardships.^13^ In light of this, the government relied on less strict behavioural change containment measures, namely wearing face masks, practising social distancing, avoiding crowded places, washing hands frequently, disinfecting rooms and surfaces and limiting times for opening shops and other business places such as markets and taverns. These containment measures have the potential to limit social and economic activities.^12^ The implication is that the longer it takes to control the pandemic, the longer the country’s economy and the health system will remain dysfunctional, leading to worsening poverty and possibly loss of lives of the greater population. Furthermore, despite repeated calls to adhere to these COVID-19 containment measures, very few people are practising the COVID-19 control measures^12^; for example, they continue to gather in large numbers, hence a high risk of infection and being infected. Lack of knowledge on the reasons why Malawians disregard some containment measures makes it difficult for the Malawi government and health stakeholders to effectively deploy containment measures that are more appropriate and appealing to the people.

## Research methods and design

This cross-sectional study was conducted in some districts in the Northern Region of Malawi. However, this paper focuses only on two rural areas, namely Nkhata Bay and Rumphi, targeting rural communities. Rumphi district has the majority of its population living in remote areas largely deprived of access to health services, TV and mobile phones. Rumphi district also borders Zambia to the North West, where unchartered routes between the district and Zambia are numerous and institutionalised, leaving the district highly exposed to foreign COVID-19 transmissions. Relatedly, the majority of the population in Nkhata Bay lives in rural areas where access to health services, TV and radio remains problematic.

The sample size was calculated with the Yamane formula as 263. A questionnaire was used to collect data from participants comprising mostly closed-ended questions. The questionnaire was translated into various local languages. The questionnaire addressed eight COVID-19 containment measures, namely wearing masks, using sanitisers, washing hands with soap, avoiding crowded places, practising social distancing and no longer shaking hands, going to funerals or going to church. Four research assistants, who are all health sciences students, were recruited to help collect the data.

The Statistical Package for the Social Sciences (SPSS) version 22 (IBM Corporation, Armonk, New York, United States) was used to capture raw data. Descriptive statistics in form of frequency analysis was performed to summarise the data in tables and figures. The chi-square (*χ*^2^) test in cross-tabulation was performed to determine the association between demographic variables and survey responses. Specifically, the researchers performed chi-square (*χ*^2^) tests to establish the relationship between the demographics (district, gender, age, household size, level of education and monthly income) and COVID-19 prevention measures (wearing masks, using sanitisers, washing hands with soap, avoiding crowded places, practising social distancing and no longer shaking hands, going to funerals or going to church).

### Ethical considerations

The study protocol was vetted and cleared by the Mzuzu University Research Ethics Committee (MZUNIREC), which is sanctioned by the National Commission for Science and Technology, which is a government agency mandated to oversee research activities, including ethics clearance and enforcement, in Malawi. The ethical certificate number is MZUNIREC/DOR/20/03. All participants signed informed consent forms and had the right to withdraw from the study at any time if they so wished.

## Results

Of the 263 participants enrolled in the study, 131 (49.8%) were from Rumphi and 132 (50.2%) from Nkhata Bay. There were more female (57.8%) than male participants (42.2%). The majority (54.4%) were in the age range of 18–29. More participants indicated living in a household size of less than five members (55.5%). Many participants (41.8%) had only a Primary School Certificate of Education (PSCE) as their highest qualification. The majority of the participants reported earning less than MK50 000.00 (83.3%). Additional details are presented in Table 1.

**TABLE 1:** Demographic characteristics.

Variable	*n*	%
**District**		
Rumphi	131	49.8
Nkhata Bay	132	50.2
**Gender**		
Male	111	42.2
Female	152	57.8
**Age**		
18–29	143	54.4
30–49	77	29.3
50 +	39	14.8
**Household size**		
Less than 5	146	55.5
Above 5 but less than 10	106	40.3
Above 10	9	3.4
**Qualifications**		
Primary School Certificate of Education	110	41.8
Junior Certificate of Education	62	23.6
Malawi School Certificate of Education	34	12.9
Diploma	4	1.5
Bachelor’s degree	1	0.4
Other qualifications	52	19.8
**Monthly income**		
Less the MK50000.00	219	83.3
Above MK50000.000 but less than MK100 000.00	24	9.1
Above MK100000.00 but less than MK200000.00	9	3.4
Above MK200 000.00 but less than MK500 000.00	7	2.7
Above MK500000.00	4	1.5

The associations between demographic variables and adherence to COVID-19 containment measures are presented in Table 2.

**TABLE 2:** Association between demographic variables and adherence to COVID-19 containment measures.

Variable	Wearing masks			Using sanitisers			Washing hands with soap			Avoiding crowded places			Practising social distance			Stopped shaking hands			Stopped going to funerals			Stopped going to church		
	*n*	%	*p*	*n*	%	*p*	*n*	%	*p*	*n*	%	*p*	*n*	%	*p*	*n*	%	*p*	*n*	%	*p*	*n*	%	*p*
**District**																								
Nkhata Bay (*n* = 131)	102	77.3	**0.01**	27	20.5	**0.03**	112	85.5	**0.35**	40	30.3	**0.68**	56	42.4	**0.00**	113	85.6	**0.48**	23	17.4	**0.51**	17	12.9	**0.02**
Rumphi (*n* = 132)	118	90.1		42	32.1		117	89.3		36	27.5		86	65.6		229	87.1		27	20.6		31	23.7	
**Gender**																								
Male (*n* = 111)	88	79.3	**0.10**	36	32.4	**0.05**	95	86.4	**0.67**	36	32.4	**0.34**	68	61.3	**0.04**	90	81.1	**0.013**	18	16.2	**0.32**	22	19.8	**0.57**
Female (*n* = 152)	132	86.8		33	21.7		134	88.2		40	26.3		74	48.7		139	91.4		32	21.1		26	17.1	
**Age**																								
18–29 (*n* = 143)	119	83.2	**0.95**	45	31.5	**0.10**	121	85.2	**0.48**	53	37.1	**0.00**	81	56.6	**0.56**	121	84.6	**0.42**	32	22.4	**0.10**	31	21.7	**0.11**
30–49 (*n* = 77)	65	84.4		16	20.8		70	90.9		14	18.2		39	50.6		70	90.9		13	16.9		12	15.6	
50 + (*n* = 39)	32	82.1		6	15.4		34	87.2		7	17.9		19	48.7		34	87.2		3	7.7		3	7.7	
**Household size**																								
Less than 5 (*n* = 146)	119	81.5	**0.01**	34	23.3	**0.42**	124	85.5	**0.04**	41	28.1	**0.93**	79	54.1	**0.99**	125	85.6	**0.63**	29	19.9	**0.81**	31	21.2	**0.39**
> 5 but < 10 (*n* = 106)	95	89.6		32	30.2		98	92.5		31	29.2		57	53.8		95	89.6		20	18.9		16	15.1	
Above 10 (*n* = 9)	5	55.6		3	33.3		6	66.7		31	29.2		5	55.6		8	88.9		1	11.1		1	11.1	
**Qualifications**																								
PSCE (*n* = 110)	98	89.1	**0.01**	24	21.8	**0.00**	97	88.2	**0.29**	31	28.2	**0.01**	61	55.5	**0.07**	96	87.3	**0.68**	19	17.3	**0.12**	16	14.5	**0.16**
JCE (*n* = 62)	50	80.6		27	43.5		54	88.5		22	35.5		36	58.1		56	90.3		19	30.6		18	29.0	
MSCE (*n* = 34)	32	94.1		13	38.2		31	91.2		16	47.1		23	67.6		27	79.4		6	17.6		7	20.6	
Diploma (*n* = 4)	2	50.0		1	25.0		2	50.0		1	25.0		1	25.0		3	75.0		0	0.0		0	0.0	
Bachelor’s degree (*n* = 1)	1	100.0		0	0.0		1	100.0		0	0.0		1	100.0		1	100.0		0	0.0		0	0.0	
PSCE dropouts (*n* =)	37	71.2		4	7.7		44	84.6		6	11.5		20	38.5		46	88.5		6	11.5		7	13.5	
**Monthly income MK**																								
< 50 (*n* = 219)	181	82.6	**0.59**	49	22.4	**0.01**	191	87.6	**0.36**	62	28.3	**0.30**	118	53.9	**0.44**	191	87.2	**0.36**	43	19.6	**0.60**	43	19.6	**0.59**
> 50 & < 100 (*n* = 24)	21	87.5		10	41.7		22	91.7		6	25.0		11	45.8		22	91.7		4	16.7		3	12.5	
> 100 & < 200 (*n* = 9)	7	77.8		4	44.4		6	85.7		5	55.6		7	77.8		6	66.7		0	0.0		1	11.1	
> 200 & < 500 (*n* = 7)	7	100.0		5	71.4		6	85.7		1	14.3		3	42.9		6	85.7		2	28.6		0	0.0	
> 500 (*n* = 4)	4	100.0		1	25.0		4	100.0		2	50.0		3	75.0		4	100.0		1	25.0		1	25.0	

MK, Malawian kwacha; JCE, Junior Certificate of Education; MSCE, Malawi School Certificate of Education; PSCE, Primary School Certificate of Education.

At the district level, a significant association was found between the district and the following COVID-19 prevention measures: wearing masks (*p* < 0.01), whereby more participants (90.1%) from Rumphi were more likely to use masks than participants from Nkhata Bay (77.3%); use of sanitisers (*p* = 0.03), whereby more participants (32.1%) from Rumphi were more likely to use sanitisers than those from Nkhata Bay (20.5%); social distancing (*p* = 0.01), whereby more participants (65.4%) from Rumphi were more likely to practise social distancing than those from Nkhata Bay (42.4%); and going to church (*p* = 0.02), whereby 23.7% participants from Rumphi were more likely to stop going to church than those from Nkhata Bay (12.9%).

Gender was significantly associated with the use of sanitisers (*p* = 0.05), whereby more men (32.4%) were more likely to use sanitisers than women (21.7%), and social distancing (*p* = 0.04), where more men (61.3%) were more likely to practise social distancing than women (48.7%).

Age was only associated with avoiding crowded places (*p* < 0.01), whereby those aged between 18 and 29 (37.1%) were more likely to avoid going to crowded places than those aged 30 and 49 (18.2%) and those above 50 (17.9%).

Household size was significantly associated with the dimensions of the use of sanitisers (*p* < 0.01) and washing hands with soap (*p* = 0.04).

Level of education affected three preventive measures significantly: Those with advanced qualifications such as a bachelor’s degree (100%) were more likely to wear masks than those with lower qualifications such as primary school dropouts (71.2%) (*p* < 0.01); those with a JCE were more likely to use sanitisers than primary school dropouts (7.7%) (*p* < 0.01); and those with an MSCE (47.1%) were more likely to avoid crowded places than those with the lowest qualification, i.e. primary school dropouts (11.5%) (*p* < 0.01).

It was further found those earning more money, such as up to MK500 000.00 (71.4%), were more likely to use sanitisers than those earning less than MK50 000.00 (22.4%) (*p* < 0.01).

Table 3 shows the associations between demographics and factors affecting the COVID-19 fight. More participants from Nkhata Bay (75%) lacked healthy literacy than those from Rumphi (65%) (*p* < 0.01), and more participants from Nkhata Bay (52.3%) than those from Rumphi (27.5%) were less knowledgeable of English and Chichewa languages (*p* < 0.01). We found that more females (59.9%) lacked access to the radio than men (46.8%) (*p* = 0.04). Older participants were more affected by fake news (*p* = 0.03), and those aged above 40 and 50 were more affected by culture than those aged between 18 and 39 (*p* = 0.07).

**TABLE 3:** Association between demographics and factors affecting COVID-19 fight.

Demographics	Lack of health literacy on COVID-19			Unavailability of free COVID-19 health services			My education level is too low to understand COVID-19 issues			Lack of resources necessary for preventing COVID-19			Lack of access to the radio through which COVI-19 messages are communicated			Poor skills in English and Chichewa languages, through which COVI-19 messages are communicated			Fake news, misinformation and myths about COVID-19			My culture forces me to disregard some COVID-19 containment measures		
	*n*	%	*p*	*n*	%	*p*	*n*	%	*p*	*n*	%	*p*	*n*	%	*p*	*n*	%	*p*	*n*	%	*p*	*n*	%	*p*
**District**																								
Nkhata Bay (*n* = 131)	100	75.8	**0.00**	70	53.4	0.32	72	54.5	0.54	93	70.5	0.51	70	53.0	0.66	69	52.3	**0.00**	89	67.4	0.51	106	80.3	0.46
Rumphi (*n* = 132)	74	56.5		61	46.6		66	50.4		87	66.4		73	55.7		36	27.5		85	64.9		100	76.3	
**Gender**																								
Male (*n* = 111)	75	67.6	0.68	61	55.5	0.13	62	55.9	0.35	73	65.8	0.43	52	46.8	**0.04**	48	43.2	0.35	67	60.4	**0.09**	86	77.5	0.78
Female (*n* = 152)	99	65.1		70	46.1		76	50.0		107	70.4		91	59.9		57	37.5		107	70.4		120	78.9	
**Age**																								
18–29 (*n* = 143)	99	69.2	0.22	73	51.4	0.72	80	55.9	0.24	94	65.7	0.21	80	55.9	0.95	59	41.3	0.37	87	60.8	**0.03**	108	75.5	**0.07**
30–49 (*n* = 77)	50	64.9		38	49.4		39	50.6		56	72.7		38	49.4		33	42.9		55	71.4		61	79.2	
50 + (*n* = 39)	23	59.0		19	48.7		18	46.2		29	74.4		23	59.0		12	30.8		30	76.9		35	89.7	
**Household size**																								
Less than 5 (*n* = 146)	93	63.7	0.60	75	51.7	0.59	78	53.4	0.72	97	66.4	0.42	80	54.8	0.45	56	38.4	0.25	94	64.4	0.78	109	74.7	0.18
> 5 but < 10 (*n* = 106)	74	69.8		52	49.1		53	50.0		74	69.8		60	56.6		43	40.6		73	68.9		88	83.0	
Above 10 (*n* = 9)	5	55.6		4	44.4		5	55.6		7	77.8		2	22.2		6	66.7		5	55.6		7	77.8	
**Qualifications**																								
PSCE (*n* = 110)	75	68.2	**0.09**	56	51.4	0.36	65	59.1	0.26	74	67.3	0.14	66	60.0	**0.02**	48	43.6	**0.03**	80	72.7	0.65	91	82.7	0.94
JCE (*n* = 62)	37	59.7		32	51.6		29	46.8		40	64.5		29	46.8		16	25.8		36	58.1		43	69.4	
MSCE (*n* = 34)	19	55.9		18	52.9		17	50.0		22	64.7		14	41.2		8	23.5		20	58.8		25	73.5	
Diploma (*n* = 4)	2	50.0		2	50.0		1	25.0		1	25.0		0	0.0		1	25.0		2	50.0		4	100.0	
Bachelor’s degree (*n* = 1)	0	0.0		0	0.0		0	0.0		1	100.0		0	0.0		0	0.0		0	0.0		1	100.0	
PSCE dropouts (*n* = 52)	41	78.8		23	44.2		26	50.0		42	80.8		34	65.4		32	61.5		36	69.2		42	80.8	
**Monthly income MK**																								
< 50 (*n* = 219)	146	66.7	**0.21**	108	49.5	0.58	115	52.5	0.99	158	72.1	**0.00**	121	55.3	0.93	91	41.6	**0.06**	145	66.2	0.39	169	77.2	0.76
> 50 & < 100 (*n* = 24)	19	79.2		13	54.2		13	54.2		16	66.7		13	54.2		12	50.0		18	75.0		20	83.3	
> 100 & < 200 (*n* = 9)	4	44.4		3	33.3		4	44.4		5	55.6		4	44.4		1	11.1		6	66.7		7	77.8	
> 200 & < 500 (*n* = 7)	3	42.9		4	57.1		4	57.1		1	14.3		3	42.9		1	14.3		4	57.1		6	85.7	
> 500 (*n* = 4)	2	50.0		3	75.0		2	50.0		0	0.0		2	50.0		0	0.0		1	25.0		4	100.0	

MK, Malawian kwacha; JCE, Junior Certificate of Education; MSCE, Malawi School Certificate of Education; PSCE, Primary School Certificate of Education; COVID-19, coronavirus disease 2019.

Participants with lower qualifications were more likely to be health illiterate than those with higher qualifications (*p* = 0.09); those with lower qualifications had less access to the radio (*p* = 0.02), and those with lower qualifications had less knowledge in English and Chichewa (*p* = 0.03). We found that participants with lower monthly incomes were more likely to lack these resources (*p* < 0.01). We found that household size was not significantly associated with any factor (*p* > 0.05).

## Discussion

With the larger rural Malawian population having limited access to the COVID-19 vaccine, following WHO COVID-19 prevention measures is heralded as key in the fight against the epidemic.^14^ This study investigated COVID-19 prevention practices among people living in rural communities in the Northern region of Malawi.

The study revealed that wearing masks, using sanitisers, practising social distancing and going to church were associated with the district; it was found that participants from the Rumphi district were more likely to follow these COVID-19 preventive measures than those from the Nkhata Bay district. This could be attributed to the level of intensity of civic education carried out by the two district hospitals – it might happen that Rumphi Hospital is more vigorous in its COVID-19 awareness campaign than Nkhata Bay. Awareness of the dangers of COVID-19 and its preventive measures has proven to reduce the transmission of the pandemic in Uganda.^15^ Based on these findings, there is need for the Ministry of Health (MoH) and its stakeholders in the COVID-19 fight not to relax their efforts in Rumphi while upscaling these efforts in the Nkhata Bay district.

It was further found that gender (in favour of men) significantly affected the use of sanitisers and social distancing. In Malawi, men are the source of income; hence, they are more likely to find the money for buying sanitisers than women, who are mostly at the receiving end of all economic misfortunes. These results are consistent with those of a study in Germany which showed that the COVID-19 pandemic affected women more heavily than men in various ways which, among others, were a result of the gender economic gap in favour of men.^16^ A report commissioned by the World Bank indicates that in lower-income countries, women are largely engaged in informal work,^17^ implying they are economically disadvantaged and find it difficult to access COVID-19 personal protective equipment (PPE); hence, it is not surprising that they could not access sanitisers. There have been anecdotal reports in Malawi that more men die of COVID-19 than women, and this information may have influenced more men to follow preventive measures than women. Much as there is no scientific evidence in Malawi, the literature suggests that across countries and age groups, once infected with COVID-19, men are more likely to die from or with COVID-19 than women.^17,18^ Because it was found that gender affects the COVD-19 fight, the health stakeholders need to take gender into account by ensuring that women and girls are included in education, decision-making and implementation initiatives.

Younger people were more likely to avoid crowded places than older people. This can be attributed to the fact that older people are generally involved in fetching basic needs for their families; hence, they are more likely to be away from their homes. These basic needs are mostly accessible in places such as markets; hence, it becomes difficult for them to avoid such crowded places. Considering that older people are more likely to die of COVID-19 than young people,^17,19,20^ these results suggest that there are greater risks resulting from the pandemic that may escalate in these communities. These findings suggest the need for community health education tailored to address the needs of specific groups.

Education was found to be key in the implementation of some COVID-19 prevention measures because the more educated the participants, the more they were likely to follow some containment measures, mainly wearing masks, using sanitisers, avoiding crowded places and practising social distancing. This is not surprising because it is common knowledge that the more educated the people, the more they are likely to understand and grasp issues, and COVID-19 issues may not be exceptional. Similar findings were reported in Iran that educated groups were more likely to follow COVID-19 preventive measures than those with lower educational levels.^21^

According to the findings, the economic status of participants is key in the fight against the pandemic, because according to the study findings, the more money participants earned per month, the more they were able to use sanitisers. Unfortunately, the study findings reveal that the majority live below the poverty line and could not afford to buy PPE such as sanitisers and face masks. The impact on the economy of the COVID-19 fight has been also reported elsewhere in the world.^22,23^ For example, De Souza et al.^24^ report a positive association between higher per-capita income and the COVID-19 fight in Brazil.

The results further revealed a significant association between the participants’ district of residence and health literacy on COVID-19 – participants from the Nkhata Bay district were more likely to be affected by lack of health literacy than those from the Rumphi district. The results suggest that people in Rumphi are more exposed to health messages on COVID-19 than those in Nkhata Bay. There is a need to amplify the COVID-19 awareness education in Nkhata Bay while ensuring that they do not relax in Rumphi. Relatedly, the results established that the district of residence was significantly associated with knowledge of English and Chichewa languages. People in Nkhata Bay had poorer knowledge of English and Chichewa languages than those in the Rumphi district. English and Chichewa languages are the official and national languages, respectively, for Malawi,^25^ and health authorities mostly use these when communicating COVID-19 messages to Malawians. The unfortunate part is that people in these districts speak their local or mother languages, which are Chitumbuka and Chitonga for Rumphi and Nkhata Bay, respectively. They only learn English and Chichewa in schools, implying that those who have not been to school cannot understand, write or read these languages. According to Thompson et al.,^26^ such citizens must rely on other citizens to be accurately informed, yet this process may lead to distortion of the meaning. In China, before the COVID-19 outbreak, Putonghua was a supreme language that was key for social inclusion,^27,28^ but noting that the language proved to be a barrier to the uptake of and compliance with public health directives, the government resorted to the adoption and enforcement of a multilingual approach.^29^ Therefore, streamlining and repackaging COVID-19 messages into local languages spoken in each district will be a more effective way of ensuring that the messages achieve the intended purpose. Such a strategy has worked in some African countries such as Ghana, where the use of songs with lyrics in local languages resulted in more uptake and actual practice of COVID-19 prevention by the people.^26^ The multilingual nature of Malawi, with more than 15 indigenous languages,^25^ implies that community radio stations are better suited to transmit such messages, unlike national radio stations which mostly produce their content in Chichewa and English.

The present study found that gender was significantly associated with a lack of access to the radio: men were more likely to access the radio than women. This could be attributable to the fact that in these two districts and other parts of Malawi, men are traditionally the heads of the family and control most of the household property, including the radio; hence, the higher number of men accessing the radio according to the results is not surprising. Failure to access the radio, where most credible messages about COVID-19 are delivered, may have contributed to more women than men being exposed to fake news and misinformation on COVID-19. Iradukun et al.^30^ suggest targeted health promotion and prevention awareness, which must be directed to the vulnerable individuals, in this case women, whom the study has proven to be more prone to COVID-19 fake news and misinformation. Fake news and misinformation in this context refer to false or unverified information about COVID-19 which is intentionally or unintentionally spread by community members.

Results revealed that older people were likely to disregard some COVID-19 containment measures because of their cultural traditions. In Malawi, older people are regarded as the custodians of cultural practices which are passed from one generation to another. In most cultures in Malawi, older people failing to patronise ceremonies attached to community culture, such as funerals, are reprimanded by the communities, and punishments include being disrespected by the community. Some of these cultural activities can fuel the spread of COVID-19 because they undermine some COVID-19 containment measures such as social distancing. Moreover, commonly, culture is a sensitive issue which health stakeholders need to approach with care when communicating to the concerned communities about its role in fuelling the pandemic. Elsewhere, when it enforced the wearing of masks and social distancing to control the pandemic, the American government faced resistance and backlash from its citizens, who expressed concern, saying their freedom was being violated.^11^ The present study confirms the results from Borg^10^ and Gaygısız et al.^31^ that culture has the potential to derail the efforts towards controlling infection of pandemics. The key custodians of Malawian culture are traditional leaders, with those at the pinnacle of the hierarchy called traditional authorities (TAs), who wield substantial influence over their subordinates and subjects. There is thus the need to engage these traditional leaders on the need to temporarily adjust some cultural practices that undermine the fight against COVID-19.

The results revealed a significant association between the level of education and health literacy on COVID-19. Health literacy entails a combined set of skills that enable people to access, understand and implement health advice and make informed health decisions.^32,33,34^ As shown in this study, lack of health literacy contributes to people falling victim to fake news, misinformation and myths about COVID-19 which are detrimental to the fight against the pandemic. It is perhaps for this reason that Habersa et al.^35^ emphasise the need to anticipate and manage misinformation by engaging with various media outlets. A report commissioned by the MoH revealed that most Malawians trust health personnel as sources of accurate information on COVID-19.^36^ Unfortunately, it is common knowledge that there are few health personnel in rural areas, and they cannot adequately serve the catchment areas, which are far and wide. More worrying is that the Health Surveillance Assistants (HSAs), who are at the periphery of the health system hierarchy in Malawi, are not adequately educated, as characterised by their basic informal training in health. The current study’s results are consistent with those of McCaffery et al.,^37^ who found that people who lacked health literacy in Australia demonstrated poor understanding of COVID-19 prevention measures and were more prone to COVID-19 misinformation and fake news.

A strong association (*p* < 0.01) was found between monthly income and lack of resources for following COVID-19 containment measures. Fighting COVID-19 requires resources. People need PPE such as face masks, hand sanitisers and well-ventilated houses. Even those containment measures that may not directly need money are related to economic issues. For instance, people may not fully follow social distancing because places such as communal markets cannot be avoided because they are the hubs of community economies, thereby leaving socio-economically disadvantaged people more susceptible to the pandemic. Moreover, some previous studies have shown that social distancing is more difficult in socio-economically disadvantaged neighbourhoods where crowding results in individuals or communities to be more likely to be exposed to COVID-19.^38,39,40,41^ With most participants living below the international poverty line of $1.90 per day,^42^ the fight against the pandemic becomes more complex and problematic. The impact of the economy on the COVID-19 fight has been also reported elsewhere in the world.^22,23,24^ For example, De Souza et al.^24^ similarly found a positive association between higher per capita income and COVID-19 diagnosis in Brazil. The present study’s findings are further consistent with those reported in Hong Kong, where during the third wave of the pandemic, as the government enforced more stringent measures, the socio-economically disadvantaged people and neighbourhoods were more affected.^9^ A study commissioned by the United Nations Development Programme^12^ noted that the high poverty rates in Malawi made people more concerned about finding food than being concerned with contracting the virus. The present study’s authors therefore agree with some previous studies that concluded that to effectively contain COVID-19 transmission, policymakers need to invest more resources in these economically disadvantaged individuals and communities.^30,31^

### Limitations of the study

Participants were recruited from rural areas, and the results may not be appropriately applicable to urban populations. More so, with data collected from two districts in the Northern region, the findings may not be adequately generalised to the population of other regions of Malawi – there are diverse factors across regions, districts and communities such as culture, economy, etc.

## Conclusion

Coronavirus disease 2019 has not spared any single place, and this study finds that people living in rural communities are socially and economically disadvantaged, making them highly vulnerable to COVID-19 infection. Disparities were found in their knowledge of the language, level of education, culture, monthly income and health literacy which compel the researchers to question the deployment of unified COVID-19 containment strategies. To this end, the authors propose that health authorities should repackage the COVID-19 containment measures, prioritising and tailoring them to meet the needs of these groups. In particular, the low levels of income of the rural people and culture are some of the key factors forcing the people to neglect COVID-19 containment measures; the authors therefore propose that the government should consider extending the Emergency Cash Transfer Programme to rural people. With the Malawian economy suffering a massive nosedive resulting from the effects of COVID-19, there is a need to adopt all possible strategies that will help control the pandemic. The researchers noted that the economy frustrates rural people from adhering to some COVID-19 containment measures such as the use of sanitisers. Encouraging rural dwellers to follow COVID-19 containment measures that have little or no economic ramifications could be a more appealing strategy. Like other previous studies, this study found that gender influences adherence to some COVID-19 containment measures; hence, the authors call upon health stakeholders to include women and girls in COVID-19 interventions. The researchers are optimistic that their findings have implications for controlling current and future communicable diseases – the strategies they have proposed might be employed in fighting such pandemics.
